# Lipid Metabolic Reprogramming in Hepatocellular Carcinoma

**DOI:** 10.3390/cancers10110447

**Published:** 2018-11-15

**Authors:** Hayato Nakagawa, Yuki Hayata, Satoshi Kawamura, Tomoharu Yamada, Naoto Fujiwara, Kazuhiko Koike

**Affiliations:** Department of Gastroenterology, The University of Tokyo, 7-3-1 Hongo, Bunkyo-ku, Tokyo 113-8655, Japan; hayatayk728@gmail.com (Y.H.); shisato13@gmail.com (S.K.); tyism123@gmail.com (T.Y.); fujiwara5358@gmail.com (N.F.); kkoike-tky@umin.ac.jp (K.K.)

**Keywords:** metabolic reprogramming, hepatocellular carcinoma, non-alcoholic fatty liver disease, obesity, fatty acid β-oxidation, carnitine palmitoyltransferase 2

## Abstract

Metabolic reprogramming for adaptation to the local environment has been recognized as a hallmark of cancer. Although alterations in fatty acid (FA) metabolism in cancer cells have received less attention compared to other metabolic alterations such as glucose or glutamine metabolism, recent studies have uncovered the importance of lipid metabolic reprogramming in carcinogenesis. Obesity and nonalcoholic steatohepatitis (NASH) are well-known risk factors of hepatocellular carcinoma (HCC), and individuals with these conditions exhibit an increased intake of dietary FAs accompanied by enhanced lipolysis of visceral adipose tissue due to insulin resistance, resulting in enormous exogenous FA supplies to hepatocytes via the portal vein and lymph vessels. This “lipid-rich condition” is highly characteristic of obesity- and NASH-driven HCC. Although the way in which HCC cells adapt to such a condition and exploit it to aid their progression is not understood, we recently obtained new insights into this mechanism through lipid metabolic reprogramming. In addition, accumulating evidence supports the importance of lipid metabolic reprogramming in various situations of hepatocarcinogenesis. Thus, in this review, we discuss the latest findings regarding the role of FA metabolism pathways in hepatocarcinogenesis, focusing on obesity- and NASH-driven lipid metabolic reprogramming.

## 1. Introduction

Hepatocellular carcinoma (HCC) is the second most frequent cause of cancer-related death worldwide [1]. Recent advances in early diagnosis and treatment have improved the short-term prognosis of patients with HCC, but the long-term prognosis remains poor even after curative treatment [2,3,4]. More than 90% of HCCs develop in the context of chronic liver damage and inflammation, and chronic hepatitis B virus or hepatitis C virus infections are typically the major catalysts [5]. However, the incidence of nonviral HCC is rapidly increasing, especially in developed countries. Most of these patients are obese, show symptoms of metabolic syndrome and suffer from nonalcoholic steatohepatitis (NASH), which is characterized by liver steatosis, inflammation, hepatocellular injury, and fibrosis [6,7]. Additionally, obesity increases the risk of HCC development in patients with viral hepatitis [8,9]. Although the mechanism by which obesity promotes hepatocarcinogenesis remains incompletely understood, various tumor microenvironmental factors, such as insulin resistance-mediated hyperinsulinemia, elevated proinflammatory cytokines induced by oxidative and endoplasmic reticulum (ER) stress, dysregulation of adipokines, and altered gut microbiota, are believed to coordinately promote HCC development [6,10,11,12,13,14,15]. Furthermore, recent studies have shown that cancer cell-intrinsic metabolic alterations, so-called metabolic reprogramming, are involved in enhanced carcinogenesis in obesity.

Cellular adaptations caused by metabolic reprogramming can be clonally selected during tumorigenesis [16]. Metabolites generated by metabolic reprogramming also exert cancer-promoting functions by modulating signaling pathways, epigenetic states, and cellular differentiation. The most well-studied metabolic change is the Warburg effect, in which cancer cells use aerobic glycolysis instead of mitochondrial oxidative phosphorylation as building blocks for cellular proliferation, leading to increased lactate production [17]. Another well-analyzed metabolic alteration is increased glutamine metabolism, referred to as glutaminolysis, which generates higher levels of α-ketoglutarate and citrate to support the activity of the mitochondrial tricarboxylic acid (TCA) cycle [18]. Furthermore, although alterations in fatty acid (FA) metabolism in cancer cells have received less attention, recent studies have revealed the importance of lipid metabolic reprogramming in carcinogenesis [19]. FAs function as signaling molecules, storage compounds, and energy sources, as well as structural components of the cell membrane, all of which are essential for cancer cell proliferation. Whereas normal cells preferentially use circulating exogenous lipids, cancer cells, including HCC cells, show a high rate of de novo lipid synthesis [20]. Furthermore, cellular uptake of FAs and fatty acid β-oxidation (FAO) is also increased in several types of cancer [21,22]. Therefore, enzymes of FA pathways have attracted attention as a potential therapeutic target [23].

Obese persons exhibit an increased intake of dietary FAs accompanied by enhanced lipolysis of visceral adipose tissue due to insulin resistance, resulting in enormous exogenous FA supplies to hepatocytes via the portal vein and lymph vessels. This “lipid-rich condition” is highly characteristic of obesity-driven liver cancer. Although the way in which HCC cells adapt to this condition and exploit it to aid their progression is not understood, we recently provided new insights into this debate through lipid metabolic reprogramming [24]. In addition, accumulating evidence supports the importance of lipid metabolic reprogramming in various aspects of hepatocarcinogenesis such as adaption to a hypoxic environment and maintenance of cancer stem cells. Thus, in this review, we discuss the latest findings regarding the roles of FA metabolism pathways in hepatocarcinogenesis, focusing on obesity- and NASH-driven lipid metabolic reprogramming.

## 2. Intracellular Pathways of FA Metabolism

**Figure 1:**
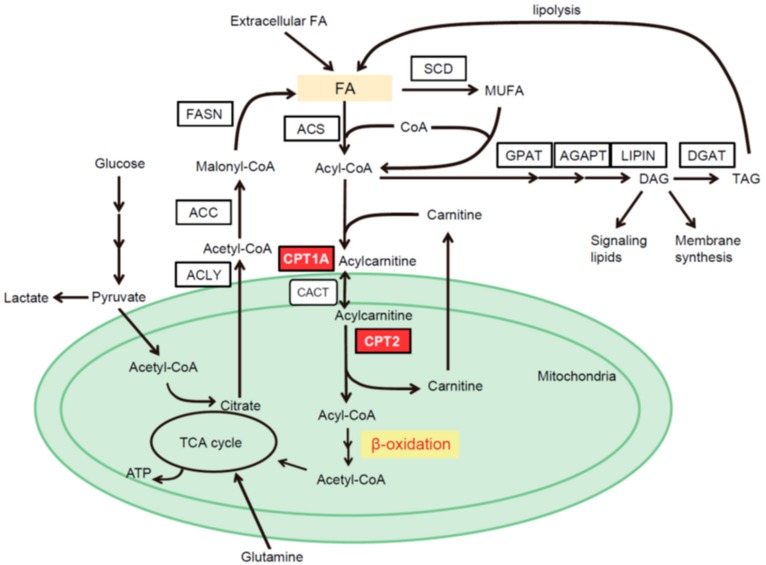
The pathways of lipid metabolism. FA: fatty acid; ACLY: ATP citrate lyase; ACC: acetyl-CoA carboxylase; FASN: fatty acid synthase; ACS: acyl-CoA synthetase; CPT1A: carnitine palmitoyltransferase 1A; CPT2: carnitine palmitoyltransferase 2; CACT: carnitine acylcarnitine translocase; SCD: stearoyl-CoA desaturase; GPAT: glycerol-3-phosphate acyltransferase; AGPAT: acylglycerolphosphate acyltransferase; MUFA: monounsaturated fatty acid; DGAT: diacylglycerol acyltransferase; DAG: diacylglycerol; TAG: triacylglycerol; TCA: tricarboxylic acid.

FAs are taken up from an extracellular source through membrane-associated transport proteins, including fatty acid transport protein-1 (FATP1), fatty acid translocase (FAT/CD36), and liver fatty acid binding protein (L-FABP), or are produced through de novo FA synthesis. The de novo FA synthesis pathway converts citrate to acetyl-coenzyme A (CoA), malonyl-CoA and, eventually, bioactive FAs through multiple enzymatic reactions catalyzed by ATP citrate lyase (ACLY), acetyl-CoA carboxylase (ACC) and fatty acid synthase (FASN) (Figure 1). FAs produced by de novo lipogenesis are saturated and converted to monounsaturated fatty acids (MUFAs), the preferred substrates for triacylglycerol (TAG) generation, by stearoyl-CoA desaturase (SCD) [19,25]. The expression levels of these enzymes are mainly regulated by sterol regulatory element-binding protein 1 (SREBP-1), a master transcription factor of lipogenesis [26]. To enter bioactive pools, exogenous or de novo synthesized FAs are activated by acyl-CoA synthetase (ACS), which converts free FAs to acyl-CoA. Acyl-CoA is converted into TAG through glycerol-3-phosphate acyltransferase (GPAT), acylglycerolphosphate acyltransferase (AGPAT), phosphatidic acid phosphohydrolase (PAP or lipin), and diacylglycerol acyltransferase (DGAT) [25]. TAG is then stored in lipid droplets as an energy source that can be degraded by lipases to release FAs [27]. Diacylglycerol (DAG), which is synthesized from phosphatidic acid during TAG synthesis, is utilized as a signaling molecule that activates protein kinase C [28]. When FAs undergo β-oxidation to generate an energy source, acyl-CoA is converted to acylcarnitine via its conjugation to carnitine by carnitine palmitoyltransferase 1 (CPT1). Then, acylcarnitine is translocated to the mitochondria via carnitine acylcarnitine translocase (CACT) and converted back to acyl-CoA by CPT2, then entering the FAO pathway, followed by the TCA cycle (Figure 1).

Whereas most normal cells prefer exogenous FA sources, cancer cells often exhibit a shift toward de novo FA synthesis. Furthermore, cancer cells sometimes use an enhanced FAO pathway, even in cells exhibiting high lipogenic activity [21]. Therefore, blocking these pathways may constitute new therapeutic strategies against cancers, including HCC. However, recent studies have shown that altered patterns of FA metabolism differ depending on the type of cancer and the tumor and host microenvironment. Therefore, in the subsequent sections, we discuss the roles of FA metabolism pathways in HCC under various situations such as obesity and hypoxic environment.

## 3. Roles of the FA Biosynthesis Pathway in HCC

A recent study analyzing the global gene expression profile of HCC revealed that genes involved in FA biosynthesis are universally upregulated in HCC compared to noncancerous liver tissues [29]. In this section, we review the roles of the major lipogenic enzymes, as well as their master regulator, SREBP-1, which are involved in hepatocarcinogenesis.

### 3.1. ACC

ACC converts acetyl-CoA to malonyl-CoA as the first rate-limiting step in de novo lipogenesis. ACC has two isoforms, ACC1 and ACC2. ACC1 catalyzes the formation of malonyl-CoA and maintains the regulation of FA synthesis, whereas ACC2 is localized in the mitochondria and mainly regulates FAO by malonyl-CoA-mediated inhibition of CPT1. Their enzymatic activities can be inhibited by AMP-activated protein kinase (AMPK)-mediated phosphorylation. Recently, ACC has attracted attention as a therapeutic target for NASH, and an ACC inhibitor that inhibits both ACC1 and ACC2, GS-0976, was reported to reduce hepatic steatosis and fibrosis markers in patients with NASH in a phase 2 clinical trial [30]. In regard to HCC, Wang et al. reported that ACC1 was an independent prognostic indicator for HCC patients, and ACC1-driven de novo FA synthesis promotes the survival of HCC cells, especially under metabolic stress conditions such as glucose limitation or antiangiogenetic treatment [31]. Furthermore, the inhibition of ACC1 and ACC2 activities by ND-654, which mimics the effects of ACC phosphorylation, suppresses chemical carcinogen diethylnitrosamine (DEN)-induced HCC development in rats [32]. On the other hand, Nelson et al. reported that mice with liver-specific ACC1 and ACC2 double knockout exhibited an increased HCC development induced by DEN [33]. Double knockout of ACC1 and ACC2 significantly enhanced antioxidant defense in the liver, which resulted in increased survival of DEN-damaged cells and eventually led to enhanced carcinogenesis. Indeed, in some human HCCs, the Nrf2-Keap1 antioxidant pathway is enhanced to eliminate reactive oxygen species (ROS) that are harmful to survival [34]. These findings suggest that the roles of ACC in HCC development may differ depending on isoforms, phosphorylation status, carcinogenesis step, and microenvironment. However, further studies are needed for validation.

### 3.2. FASN

FASN, the most extensively investigated lipogenic enzyme in cancer, is responsible for the synthesis of palmitate (C16:0) from acetyl-CoA and malonyl-CoA in the presence of NADPH [35]. FASN is frequently upregulated in various cancers, and its increased expression is associated with chemoresistance, metastasis, and poor prognosis. Several studies have shown that knockdown or pharmacological inhibition of FASN suppressed the growth of HCC in vitro [36,37]. In vivo, overexpression of an activated form of Akt by hydrodynamic tail vein injection induced HCC accompanied by enhanced de novo lipogenesis in mice [36], and genetic ablation of FASN completely suppressed Akt-driven HCC development through the inhibition of Rictor/mTORC2 signaling [38]. The expression of FASN is also regulated at the post-translational level through acetylation-mediated proteasomal degradation. FASN acetylation is frequently reduced in human HCC, which correlates with increased histone deacetylase 3 (HDAC3). An HDAC3 inhibitor destabilizes FASN proteins and suppresses the growth of HCC [39]. FASN inhibitors are currently being studied preclinically, and are beginning to appear in human trials. TVB-2640 is the most recent orally available FASN inhibitor and is currently in clinical trials for the treatment of solid tumors [40,41]. However, since several first-generation FASN inhibitors exhibited strong toxicity in preclinical and clinical trials [35], targeting HDAC3 may provide a new strategy to in turn target FASN. However, it should be noted that FASN is not exclusively the target of HDAC3.

### 3.3. SCD

SCD catalyzes the insertion of a cis double bond at the delta-9 position in saturated FAs and generates MUFAs, mainly palmitoleate (C16:1) and oleate (C18:1) from palmitate (C16:0) and stearate (C18:0), respectively. Whereas four SCD isoforms (SCD1–SCD4) have been identified in mice, only two isoforms (SCD1 and SCD5) have been identified in humans, with human SCD1 being co-orthologous to the four murine isoforms. Budhu et al. performed comprehensive gene expression and metabolic profiling of HCC and reported that the lipogenic network involving SCD signaling was significantly associated with HCC progression and patient outcome [20]. They showed that palmitoleate (C16:1), the biological end product of SCD, promoted the migration of HCC cells, whereas knockdown of SCD in HCC cells reduced cellular migration and xenograft formation. Oleate (C18:1), another end product of SCD, also had tumor-promoting roles in HCC by phosphatase and tensin homolog deleted on chromosome 10 (PTEN) downregulation via mTOR/NF-κB-mediated upregulation of microRNA-21 [42,43]. SCD was also reported as a target of β-catenin and MUFAs produced by SCD, feeding forward to amplify the Wnt pathway by supporting the expression of the Wnt receptor LRP5/6, which maintains HCC-initiating stem-like cells [44]. MUFAs inhibited binding of Ras-related nuclear protein 1 to Transportin 1 and reduced nuclear import of ELAV-like protein 1 (HuR), which resulted in increased cytosolic levels of HuR and subsequent HuR-mediated stabilization of LRP5/6 mRNAs. Thus, the accumulation of MUFAs by enhanced SCD activity promoted hepatocarcinogenesis, and reshifting the balance of FA composition toward saturation by SCD inhibition may be a potential therapeutic strategy for HCC. Furthermore, SCD activates the unfolded protein response through ER stress, which is associated with sorafenib resistance in HCC [45], suggesting that targeting SCD in combination with sorafenib may exert a synergistic effect on HCC.

### 3.4. SREBP-1

The transcription factor SREBP-1 plays a central role in cellular FA metabolism and controls the expression of various lipogenic enzymes, including ACC, FASN, and SCD. SREBP-1 is located at the ER membrane as an inactive precursor and is transported to the Golgi apparatus by the escort protein SREBP cleavage-activating protein (SCAP) in response to insulin and sterol depletion, and is then proteolytically cleaved in the Golgi to generate an active form that activates the transcription of target genes in the nucleus [26]. SREBP-1 has two isoforms, SREBP-1a and SREBP-1c. SREBP-1c is the dominant isoform in HCC, as well as in the normal liver [46]. Large-scale gene expression profiling conducted by Yamashita et al. revealed significant activation of the SREBP-1-mediated lipogenic pathway in HCC, and showed that higher expression of SREBP-1 proteins was associated with a poor prognosis [47]. The suppression of SREBP-1 in HCC cells induced growth arrest and apoptosis, whereas the overexpression of SREBP-1 enhanced cellular proliferation, suggesting that SREBP-1 may be a therapeutic target for HCC. Although drugs directly targeting SREBP-1 have not yet been established, a recently developed small organic molecule, fatostatin, strongly inhibited SREBP-1 activation by inhibiting the ER–Golgi translocation of SREBP-1 by binding to SCAP [48]. Since liver-specific deletion of SCAP in mice significantly suppressed DEN-induced HCC development [49], targeting SCAP may be a promising alternative treatment strategy for HCC. In fact, fatostatin was recently reported to inhibit an SREBP-dependent lipogenic program that promotes metastatic prostate cancer [50]. However, SREBP-1 regulates a wide range of lipogenic genes, and fatostatin inhibits not only SREBP-1-mediated lipogenesis but also SREBP-2-mediated cholesterol synthesis. Therefore, unfavorable side effects should be noted.

Several chemical inhibitors targeting FA biosynthesis pathways discussed in this section are currently in preclinical and clinical trials for cancer treatment. We summarized these inhibitors in Table 1.

## 4. Lipid Metabolic Reprogramming in HCC in Response to Dyslipidemia Associated with Obesity or NASH

### 4.1. CPT2 Downregulation-Mediated Lipid Metabolic Reprogramming in Obesity- and NASH-Driven HCC

A lipid-rich condition, especially a non-esterified FAs-rich condition, is a characteristic environment for obesity- and NASH-driven HCC [59], but how malignant cells adapt to such an environment and exploit it to aid their progression is not yet understood. A recently described histological variant of HCC, steatohepatitic HCC (SH-HCC), which is characterized by large droplet steatosis and ballooning in tumor cells, pericellular fibrosis, and inflammation, has been reported to be associated with underlying steatohepatitis, with or without viral hepatitis infection [60,61]. Importantly, various mouse obesity- and NASH-driven HCC models also exhibit more prominent tumor steatosis than nontumor hepatocytes [6,10,42], suggesting that the marked steatosis of tumor cells may be representative of the characteristically altered metabolism seen in obesity-driven HCC, and thus a key phenotype linking obesity, NASH, and HCC. Based on these findings, we recently identified characteristic metabolic changes in obesity-driven HCC through comprehensive analyses of metabolomics profiles using mouse HCC samples [24], as discussed below.

**Figure 2:**
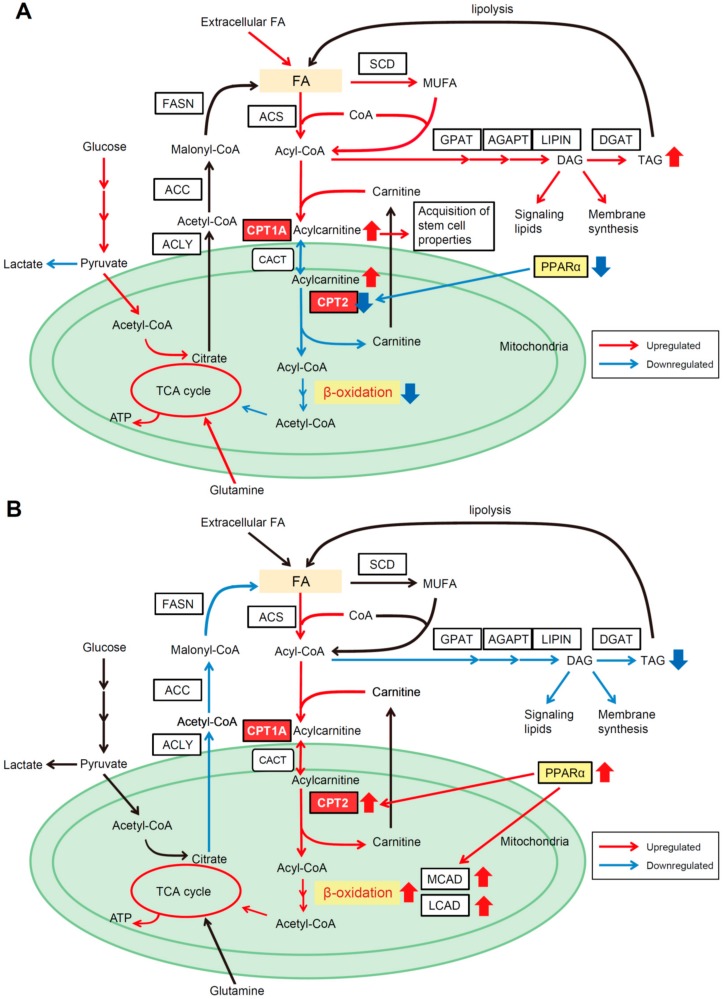
Two distinct lipid metabolic alterations in hepatocellular carcinoma (HCC). (**A**) Lipid metabolic reprograming in obesity- and nonalcoholic steatohepatitis (NASH)-related HCC. Fatty acid β-oxidation (FAO) is suppressed for adaptation to a lipid-rich environment. (**B**) Lipid metabolic reprograming in β-catenin-activated HCC. FAO is activated to fuel HCC.

We first carried out liquid chromatography–mass spectrometry (LC–MS)-based untargeted metabolomic profiling using paired nontumor and HCC tissues obtained from DEN-injected 8-month-old mice maintained on a normal diet or a high-fat diet (HFD), and found extensive accumulation of long-chain acylcarnitine species in HFD-fed HCC tissues (HFD-HCC). Among the acylcarnitine metabolism-related genes, increased expression of CPT1A (a hepatic isoform of CPT1) and ACSL4 (a member of the ACS family), and decreased expression of CACT and CPT2, were detected in HFD-HCC, indicating that despite the enhanced conversion of FAs to acylcarnitine, the reconversion of acylcarnitine to acyl-CoA was suppressed, which could account for the marked accumulation of acylcarnitine species (Figure 2A). A similar expression pattern of acylcarnitine metabolism-related genes was observed in other obesity- and NASH-driven mouse HCC models, including HFD-fed *MUP-uPA* mice [6] and *PIK3CA* transgenic mice [42]. In particular, the downregulation of CPT2 in tumor tissues was a common finding. Importantly, the expression of CPT2 was also downregulated in human SH-HCC, and NASH patients with HCC showed increased serum levels of acylcarnitine, suggesting that a similar metabolic change may occur in human obesity-mediated HCC. Consistent with our results, a recent study also showed high serum acylcarnitine levels in patients with HCC [62], suggesting that serum acylcarnitine levels may serve as a biomarker of HCC. We also conducted capillary electrophoresis–mass spectrometry (CE–MS) analysis, which revealed the suppression of FAO in HFD-HCC due to CPT2 downregulation. This could account for the marked steatotic changes in HCC. Of note, glucose was utilized for oxidative phosphorylation to compensate for suppressed FAO in HFD-HCC, unlike the Warburg effect.

We further analyzed the significance of CPT2 downregulation in obesity-mediated hepatocarcinogenesis. HCC cells in which CPT2 was knocked down acquired resistance to saturated FA-induced lipotoxicity by inhibiting excessive FAO and subsequent Src-mediated c-jun NH2-terminal kinase (JNK) activation, which plays a key role in lipotoxic cell death [63,64,65,66]. The lipotoxicity-resistant HCC cells established by chronic exposure to palmitic acid also revealed decreased CPT2 expression. Although FAO can efficiently supply energy to aid proliferation of cancer cells, excessive FAO results in excessive electron flux in the electron transport chain that can generate ROS and metabolic stress leading to cell death [67,68]. Lipotoxic hepatocyte death promotes hepatocarcinogenesis through subsequent inflammatory and regenerative responses in NASH [6]. However, HCC cells must survive in such a lipid-rich environment. Thus, CPT2 downregulation enables HCC cells to escape from lipotoxicity for adaptation to a lipid-rich environment. Furthermore, oleoylcarnitine (AC18:1), the long-chain acylcarnitine that accumulates through CPT2 downregulation-induced suppression of FAO, enhances hepatocarcinogenesis via signal transducer and activator of transcription 3 (STAT3)-mediated acquisition of stem cell properties. Altogether, CPT2 downregulation-mediated lipid metabolic reprogramming not only enables HCC cells to escape lipotoxicity, but also promotes hepatocarcinogenesis through the accumulation of acylcarnitine as an oncometabolite (Figure 2A). More recently, Lin et al. also reported that CPT2 downregulation in HCC promoted tumorigenesis and chemoresistance to cisplatin, which further supports the beneficial effects of CPT2 downregulation for hepatocarcinogenesis [69].

### 4.2. β-Catenin Determines the Dependence on FAO for HCC Development

In our previous study, we also showed that the downregulation of CPT2 in obesity- and NASH-driven HCC was, at least in part, attributed to decreased peroxisome proliferator-activated receptor alpha (PPARα). A more recent study by Senni et al. uncovered a pivotal role for β-catenin in determining which energy source to use (glycolysis or FAO) for tumor growth by regulating the expression of PPARα [70]. Enhanced FAO and reduced glycolysis accompanied by increased expression of PPARα and CPT2 were observed in β-catenin-activated HCCs derived from mice and humans. PPARα also regulated the expression of acyl-CoA dehydrogenases such as medium- and long-chain acyl-CoA dehydrogenase (MCAD and LCAD, respectively), which catabolize the initial step of FAO in the mitochondria. Genetic ablation of PPARα or inhibition of FAO by the CPT1 inhibitor etomoxir significantly blocked the development of β-catenin-activated HCC in mice, suggesting that β-catenin controls the dependence on FAO for HCC development, and that FAO is the driving force for β-catenin-activated HCC (Figure 2B). In contrast, in patients with β-catenin nonmutated HCC, the expression of CPT2 in HCC tissues was significantly lower than in adjacent nontumor tissues, consistent with another study analyzing the global gene expression profile of HCC [29]. Of note, the frequency of mutation and activation of β-catenin was significantly low in human SH-HCC and tumors arising in HFD-fed *MUP-uPA* mice [71,72]. Therefore, the absence of β-catenin activation in HCC may be advantageous for adaptation to a lipid-rich environment.

## 5. Lipid Metabolic Reprogramming in HCC for Adaption to a Hypoxic Environment

Hypoxia arises in HCCs through the accelerated growth rates of cancer cells in the absence of an efficient blood supply [73], and recent studies have shown the importance of lipid metabolic reprogramming in HCC for adaptation to a hypoxic environment.

Huang et al. reported the critical role of hypoxia inducible factor-1α (HIF-1α)-mediated suppression of FAO in the growth of HCC under hypoxic conditions [68]. HIF-1α inhibits FAO by repressing the expression of MCAD and LCAD. Since mitochondrial respiration under hypoxia results in the increased generation of ROS, which leads to growth inhibition and apoptosis of cancer cells [74], the HIF-1α-mediated suppression of FAO was beneficial for HCC cells in terms of adaptation to a hypoxic environment. In addition, inhibiting LCAD decreased the expression of PTEN and promoted the proliferation of HCC cells. Interestingly, enhanced FAO under normoxic conditions did not affect the growth of HCC. Therefore, the authors concluded that HIF-1α suppresses FAO to facilitate the progression of HCC under hypoxic conditions.

Bjornson et al. proposed another pathway to lipid metabolic reprogramming in HCC under hypoxic conditions [29]. They reconstructed a functional genome-scale metabolic model by analyzing the global gene expression profile of 361 HCC tissues and determined that tumor growth under hypoxic conditions is associated with increased expression of mitochondrial acetyl-CoA synthetase 1 (ACSS1), which converts acetate to acetyl-CoA. In their model, FA biosynthesis, pentose phosphate, and glycolysis were significantly upregulated in HCC, whereas FAO and gluconeogenesis were significantly downregulated. Conversely, the uptake of acetate and subsequent generation of acetyl-CoA from acetate by ACSS1 in the mitochondria were significantly enhanced. Mitochondrial acetate could be used as an extra carbon source to fuel the FA biosynthesis required for the proliferation of HCC cells under hypoxic conditions.

On the other hand, Iwamoto et al. recently reported that HCC cells rather utilize FAO for their survival under antiangiogenic drug-induced hypoxic conditions [75]. They showed that antiangiogenic drug-induced oxygen and nutrient depletion switched glucose-dependent metabolism to lipid-dependent metabolism by enhancing the uptake of free FAs and subsequent FAO, which stimulates cancer cell proliferation. This phenomenon was mediated by the hypoxia-induced increased phosphorylation of AMPK. Especially, metastasized HCC cells in lipid-rich local environments, such as a steatotic liver and adipose tissue, had access to easily available extracellular FAs. In addition, the CPT1 inhibitor etomoxir significantly increased the antitumor effect of antiangiogenic drugs. Since antiangiogenic drugs such as sorafenib and lenvatinib are currently the main agents used for the treatment of patients with HCC [76], elucidating the mechanisms of antiangiogenic drug resistance is very important. The combination therapy of antiangiogenesis and lipid metabolism may be a promising new strategy against HCC.

Taken together, the role of FAO in the growth and survival of HCC may be context-dependent, and more extensive investigations are needed to understand the effect of cancer metabolic reprogramming on FAO.

## 6. Lipid Metabolic Reprogramming in Cancer Stem Cells

Recent studies have suggested the existence of a subpopulation of cells within the tumor, so-called cancer stem cells (CSCs), which are typically slow-cycling, but under certain conditions capable of proliferating to self-renew and provide tumor cells. CSCs are generally resistant to chemotherapy and responsible for rapid recurrence owing to their high tumor-forming potential. In recent years, metabolic features including lipid metabolism of CSCs of various tumor types have been identified [77,78,79,80].

The expression of FASN and de novo lipogenesis are increased in glioma CSCs compared to non-CSCs [53]. FASN inhibitor cerulenin decreased markers of CSCs and suppressed proliferation and migration of glioma CSCs, suggesting that FASN-mediated lipogenesis plays a pivotal role in the maintenance of glioma CSCs. In ovarian cancer, Li et al. identified significantly increased unsaturated FAs in ovarian CSCs compared to non-CSCs using Raman microspectroscopy and mass spectrometry [56]. Increased amounts of unsaturated FAs in ovarian CSCs were caused by upregulation of SCD1, and SCD1 and NF-κB composed a positive feedback loop to maintain the high-level expression of SCD1. SCD1 inhibitor CAY10566 suppressed cancer stemness and tumor initiation capacity as well as activation of the NF-κB pathway. Noto et al. also showed the importance of SCD1 in survival and propagation of lung CSCs [57]. SCD1 activity induced the release of Wnt ligands such as Wnt3a, which in turn activated β-catenin and yes-associated protein (YAP)/ transcriptional co-activator with PDZ-binding motif (TAZ). SCD1 inhibitor MF-438 suppressed spheroid formation of lung CSCs by inhibiting activation of β-catenin and YAP/TAZ. Tirinato et al. identified accumulation of lipid droplets in colorectal CSCs using Raman microspectroscopy, and interestingly, lipid droplets might be used as a functional marker of colorectal CSCs, in addition to molecular markers [81]. Although the existence of CSCs in HCC is still a matter of some debate, Re et al. showed that loss of macroH2A1 (a variant of the histone H2A and an epigenetic regulator of stem cell function) in HCC led to CSC-like features such as chemoresistance and tumor-forming capacity through metabolic changes [82]. MacroH2A1 depletion in HepG2 cells not only enhanced lipogenesis through increased production of acetyl-CoA but also activated the pentose phosphate pathway to provide precursors for the synthesis of nucleotides. These metabolic changes were mediated by activation of the Liver X receptor (LXR) pathway, and inhibition of LXR attenuated CSCs-like features. Thus, although the metabolic features of CSCs are still poorly understood, identification of the metabolic reprogramming that occurs in CSCs may provide novel therapeutic approaches in the future.

## 7. Conclusions

Recent next-generation sequencing technology has enabled comprehensive mutational and transcriptome profiling of HCC [83,84]. However, although antiangiogenic drugs and immune checkpoint inhibitors are currently approved as molecular targeted drugs against HCC [76], they generally provide limited therapeutic benefits for survival improvement and at present there are no drugs targeting the mutation itself or mutation-associated intracellular signaling pathways. The reasons behind this may be that HCC is a heterogeneous tumor with few driver mutations, and it often arises polyclonally based on chronic liver disease. Therefore, therapies targeting the tumor microenvironment will play central roles in the treatment of advanced HCC. However, as discussed in this review, HCC exhibits several characteristic lipid metabolic changes that could serve as new therapeutic targets. In particular, the FA biosynthesis pathway is universally enhanced in HCC and could therefore be a therapeutic target. Additional lipid metabolism alterations promoting adaptation to the local environment have also been identified in HCC, which may lead to a new type of precision medicine targeting cancer-specific metabolic reprogramming. Although we focused on the role of FA metabolism in this review, FA metabolism is complexly associated with the status of other metabolic pathways, such as glucose metabolism. Thus, further studies are essential to obtain a deeper understanding of metabolic reprogramming to allow its application in clinical settings.

## Figures and Tables

**Table 1:** Drugs targeting FA biosynthesis pathways for cancer treatment.

Target	Drugs	References
ACC (acetyl-CoA carboxylase)	ND-654, TOFA	[32,51]
FASN (fatty acid synthase)	TVB-2640, Cerulenin, Orlistat, C75, Triclosan, GSK2194069, Fasnall, EGCG	[40,41,52,53]
SCD (stearoyl-CoA desaturase)	A939572, CAY10566, MF-438, BZ36	[54,55,56,57]
SREBP-1 (sterol regulatory element-binding protein)	Fatostatin, FGH10019	[50,58]
